# “One community at a time”: promoting community resilience in the face of natural hazards and public health challenges

**DOI:** 10.1186/s12889-023-17458-x

**Published:** 2023-12-14

**Authors:** Chenwei Ma, Chen Qirui, Yang Lv

**Affiliations:** 1 School of Public Administration, Sichuan University, Chengdu, 610065 China; 2 College of Teachers, Chengdu University, Chengdu, 610106 China

**Keywords:** Disaster resilience, Vulnerability, Natural hazards, Disaster preparedness, Public health, Social capital

## Abstract

**Background:**

Resilience is vital for facing natural disasters and public health challenges. Despite the significance of resilience-building activities, there is a scarcity of locally-tailored planning and response strategies, leaving communities incapable of addressing the unique challenges posed by natural disasters and public health crises. This study aims to explore how the “One Community at a Time” approach enhances community resilience in facing natural hazards and public health challenges.

**Methods:**

A systematic review was conducted over journal articles published from January 2001 to April 2023 through PRISMA approach. Multiple databases such as Web of Science and Scopus were thoroughly searched. We used independent screening by two researchers and painstaking data extraction using standardized forms. This approach was adopted to assure the reliability, validity, and precision of our study selection and analysis. The included studies’ quality was evaluated by the Mixed Methods Appraisal Tool.

**Results:**

In the evaluation, 35 studies were deemed eligible for inclusion and underwent in-depth examination. Several major components of “One Community at a Time” have been identified, including social capital and networks, local knowledge and learning, effective governance and leadership, preparedness and response capacity, and adaptive infrastructure and resources. This framework highlights the significance of individualized approaches to resilience-building initiatives, recognizing that each community has specific strengths, needs, and challenges.

**Conclusion:**

Relevant stakeholders can adapt suitable resilient strategies to help prepare and recover from natural hazards and public health challenges. By adopting a localized strategy, stakeholders can collaborate to develop a culture of readiness and resilience, ultimately leading to more sustainable and resilient communities. This framework advises community-based groups, local government, and other stakeholders on prioritizing partnerships, preparedness planning, community participation, and leadership as essential components of creating and maintaining resilience. “One Community at a Time” framework offers practical guidance for community-based organizations, local government, and other stakeholders to prioritize partnerships, preparedness planning, community participation, and leadership as essential components of creating and sustaining resilience.

**Supplementary Information:**

The online version contains supplementary material available at 10.1186/s12889-023-17458-x.

## Introduction

Natural disasters and public health issues are occurring more often and with greater severity than ever [1]. Urbanization, population increase, environmental degradation, and climate change have all escalated these problems [2]. Millions of people worldwide are more often affected by various occurrences, including hurricanes, floods, earthquakes, wildfires, and pandemics. These incidents have grave repercussions, including fatalities, evictions, economic disruptions, and long-term social and psychological effects on the impacted communities [3].

The importance of addressing these issues and fostering resilience at various levels, including local communities, has been emphasized by international initiatives like the Sendai Framework for Disaster Risk Reduction, the Paris Agreement on Climate Change, and the Sustainable Development Goals. The “One Community at a Time” concept highlights the significance of locally specialized and customized resilience-building initiatives, recognizing that each community has unique requirements and resources that must be taken into account to effectively decrease the impacts of natural catastrophes and public health crises [4]. Research [3, 5] shows that social networks, trust, and collaboration among community members are crucial for promoting community resilience. Other studies have concentrated on the value of local expertise, cultural practices, and traditional coping mechanisms in fostering resilience, contending that these elements can increase the efficacy of DRR and public health preparedness efforts [5, 6]. The “One Community at a Time” approach includes institutional and governance considerations [7]. According to studies [8, 9], community people must be involved in the development and execution of programs to enhance resilience. These procedures must be inclusive, participatory, and transparent. Additionally, early warning systems, training programs, and emergency management infrastructure are important local capabilities for preparation and response to create [10–12].

Recent research has also looked at the function of adaptable infrastructure in the “One Community at a Time” strategy [13, 14]. According to researchers, resilience concepts must be integrated into urban planning, building design, and transportation systems for communities to endure and recover from natural disasters and public health concerns [6, 15–17]. Additionally, research has pushed for the adoption of ecosystem-based approaches to disaster risk reduction (DRR) and public health preparedness, highlighting the necessity of protecting and restoring natural resources, such as forests, wetlands, and urban green spaces, to improve community resilience [6, 18, 19]. The scholarly study has increased due to the rising importance of the “One Community at a Time” concept. The role of social capital [3, 20–22], local knowledge [5, 12, 23], governance [24–27], preparedness and response capacity [28–30], and adaptive infrastructure [31–33] are just a few recent topics that have been covered in recent literature concerning this localized approach to resilience. Although these studies have shed light on important issues, it is still unclear how the “One Community at a Time” approach’s essential components may be successfully operationalized in reality [34].

Despite these advancements in the literature, there is still a need for an all-encompassing framework that can be put into practice and contains the essential components of the “One Community at a Time” method. A complete and practical framework incorporating the idea of “One Community at a Time” must include these many components including social capital and networks, local knowledge and learning, effective governance and leadership, preparedness and response capacity, adaptive infrastructure and resources. This research reveals two main gaps that are crucial in strengthening communities against the numerous challenges posed by public health crises and natural catastrophes. First, the exploration of the actual application and operationalization of the framework in real-world contexts is currently lacking in scholarly research. Although the theoretical foundations of this concept are strong, it is crucial to evaluate how these elements are expressed and interact in various community settings in order to ensure their practical effectiveness. Second, it is important to note that the current body of literature, although vast, may not necessarily offer a comprehensive perspective encompassing all the essential elements within the framework. Certain components may be inadequately represented or disregarded, resulting in possible weaknesses within the process of enhancing resilience. Therefore, this study assumes that community resilience in the face of public health challenges and natural hazards does not have locally-tailored planning and response strategies. So, it aims to address these gaps by conducting a comprehensive investigation into the operational difficulties of the framework and assuring a detailed examination of each constituent element [35]. The objective is to improve the practical applicability of the framework and guarantee that it is both theoretically robust and operationally efficient in strengthening community resilience [36].

This study identifies the important components of this localized strategy and combine them into a logical and useful framework. Therefore, a research question has been addressed in this study: How does the ‘One Community at a Time’ framework, with its emphasis on localized, community-driven initiatives, contribute to enhancing community resilience against natural hazards and public health challenges? This study attempts to provide a framework for putting the “One Community at a Time” approach to resilience-building into practice that can guide disaster risk reduction policy and practice as well as public health preparation. In doing so, we seek to add to the body of research already available on this localized approach to resilience and provide a practical tool for practitioners and policymakers in their initiatives to fortify communities in the face of natural disasters and public health issues.

The originality of this research resides in its systematic approach to bringing together the disparate and dispersed literature on the “One Community at a Time” idea. Our goal is to close the gap between theory and practice by identifying the important components of this method and incorporating them into a thorough and practical framework. Our work also adds to the expanding body of knowledge on resilience and DRR by offering a comprehensive understanding of the elements that make the “One Community at a Time” strategy successful, promoting evidence-based policymaking and planning. This study will not only add to the body of information about the “One Community at a Time” idea, but it will also provide practitioners and policymakers with a helpful tool to utilize in their attempts to fortify communities in the face of natural disasters and problems with public health.

## Methodology

### Research design

Preferred Reporting Items for Systematic Reviews and Meta-Analyses (PRISMA) is a generally accepted set of guidelines for performing and disclosing systematic reviews that guarantees rigor, openness, and repeatability [37]. The PRISMA method helps researchers conduct reviews by providing a 27-item checklist and a four-phase flow diagram.

### Research protocol

A research protocol was developed before the review began to guarantee a methodical and thorough approach. The study topic, eligibility requirements, search strategy, data sources, data extraction techniques, and quality evaluation methodologies were all included in the protocol (Table 1).

**Table 1:** Research protocol

Items	Description
Research question	How can “One Community at a Time” enhance community resilience in facing natural hazards and public health challenges?
Database	Web of Science and Scopus
Document	Only peer-reviewed articles
Language	English
Publication period	From January 2001 to April 2023
Search terms	Community resilience, One Community at a Time, resilience-building, adaptation, and recovery, natural disasters, earthquakes, hurricanes, and floods, pandemics, epidemics, outbreaks, and disease
Search fields	Title, abstract, and keywords
Inclusion criteria	The study should focus on community resilience, One Community at a Time, and public health.
Exclusion criteria	Inaccessibility of full text, doubling, and non-English articles. Furthermore, articles not focusing on tourism resilience and recovery are ignored.

### Overview of the PRISMA approach

For performing and reporting systematic reviews in the social and behavioral sciences, PRISMA is an evidence-based approach. It seeks to improve the review process’s uniformity, openness, and thoroughness [37]. Four crucial phases make up the PRISMA approach: (1) the selection of relevant studies, (2) eligibility screening, (3) evaluation of the caliber of eligible research, and (4) data extraction and synthesis. This methodology was used to guarantee a thorough and open review procedure, reducing biases and boosting the validity of the results.

## Data sources and search methodology

### Databases and search terms

The selection of Web of Science and Scopus was based on their comprehensive coverage of peer-reviewed literature in the domains of public health and disaster management. Both databases have established reputations for their stringent indexing criteria and provide a diverse range of publications that are relevant to the focus of the study. The extensive breadth of the literature evaluation guarantees that it encompasses a wide range of sources and provides a thorough and accurate representation of the current state of research in these domains. A thorough search strategy was created to find pertinent papers on the “One Community at a Time” concept for fostering resilience in the face of public health issues and natural disasters. The research question was the source of the keywords and search terms, which included words like “community resilience,” resilience-building, adaptation, and recovery, as well as words like natural disasters, earthquakes, hurricanes, and floods, as well as words like pandemics, epidemics, outbreaks, and disease. Boolean operators (AND, OR) and truncation symbols (*) were utilized to combine and sharpen search phrases. Web of Science, and Scopus databases were searched to identify most relevant documents.

The Boolean operators ‘AND’ and ‘OR’ was employed in a deliberate manner to merge search phrases and either broaden or restrict the extent of the search. For example, the conjunction ‘AND’ was used to establish a connection between distinct ideas (e.g., ‘community resilience AND natural catastrophes’), so ensuring the retrieval of articles that included both phrases. In contrast, the operator ‘OR’ was used to include articles that referenced any of the associated phrases (such as ‘earthquakes OR hurricanes OR floods’), so expanding the scope of the search to embrace a diverse range of natural catastrophes.

In the Web of Science database search, the truncation sign (*) was included in order to encompass a wide range of word ends and spellings. For example, by using the term ‘resilien*’, the search was expanded to include publications that encompassed related terms such as ‘resilience,’ ‘resilient,’ ‘resiliency,‘etc.

### Criteria for inclusion and exclusion

To guarantee a targeted and relevant evaluation, inclusion criteria were established beforehand. Studies were included if they (1) evaluated the “One Community at a Time” strategy for building and maintaining resilience in the face of natural disasters and/or public health issues, (2) used quantitative, qualitative, or mixed-methods research approaches, and (3) were published in English. Additionally, relevant documents from the reference list were retrieved using the Google search engine. Non-peer-reviewed literature, research that did not emphasize the “One Community at a Time” strategy, and studies unrelated to public health or natural hazards were also excluded.

### Study selection process

A full-text evaluation followed Title and abstract screening in the two rounds of the research selection procedure. During the systematic review process, the outcomes of the systematic searches conducted on Web of Science and Scopus were carefully uploaded to the reference management software, Mendeley. In order to uphold the credibility and precision of our assessment, we utilized the deduplication option of Mendeley to identify and eliminate duplicate entries by utilizing a predetermined set of criteria, including but not limited to the title, author, and publication year. Through the utilization of this functionality, we have secured the distinctiveness and pertinence of each document, so removed possible duplications and ensured the accuracy of the selected studies. Following the elimination of duplicates, the titles and abstracts of the found papers were independently reviewed by two reviewers using the predetermined inclusion and exclusion criteria. Discussion and, if required, interaction with a third reviewer were used to settle disagreements amongst the reviewers. Studies that satisfied the qualifying requirements were then submitted to a full-text review, with the publications being evaluated by two reviewers independently for quality and relevance. Any differences were discussed or settled in cooperation with a third reviewer.

### Synthesis and data extraction

A uniform data extraction form was used to collect the necessary data from the approved studies throughout the data extraction process. Study features (e.g., authors, publication year, study location), research design, population, intervention, significant results, and implications for the “One Community at a Time” approach to resilience-building were all included in the data that was extracted.

Two reviewers separately gathered data from the included papers, and any inconsistencies were settled by conversation or contact with a third reviewer. After data extraction, a thematic synthesis was done to find the “One Community at a Time” approach’s common themes, patterns, and critical components. To synthesize the data, it was necessary to code the extracted information, organize the codes into categories, and summarize the results under overarching themes.

### Quality evaluation

Utilizing the Mixed Methods Appraisal Tool (MMAT) [38], which is intended for evaluating the methodological quality of quantitative, qualitative, and mixed-methods investigations, the included studies’ quality was evaluated. The MMAT is divided into five areas, each of which has a set of criteria for assessing the suitability of the study design, the gathering and processing of data, and the overall rigor of the research. Each study’s quality was evaluated by two reviewers separately, with disagreements being settled by conversation or consultation with a third reviewer. The interpretation and synthesis of the results were guided by the quality evaluation procedure, with greater weight given to the synthesis of research of higher quality. The “One Community at a Time” strategy of building and maintaining resilience in the face of natural disasters and public health concerns is examined rigorously and transparently in this systematic review, which follows the PRISMA methodology.

## Results

### Systematic review results

#### Selection of documents

**Fig. 1:**
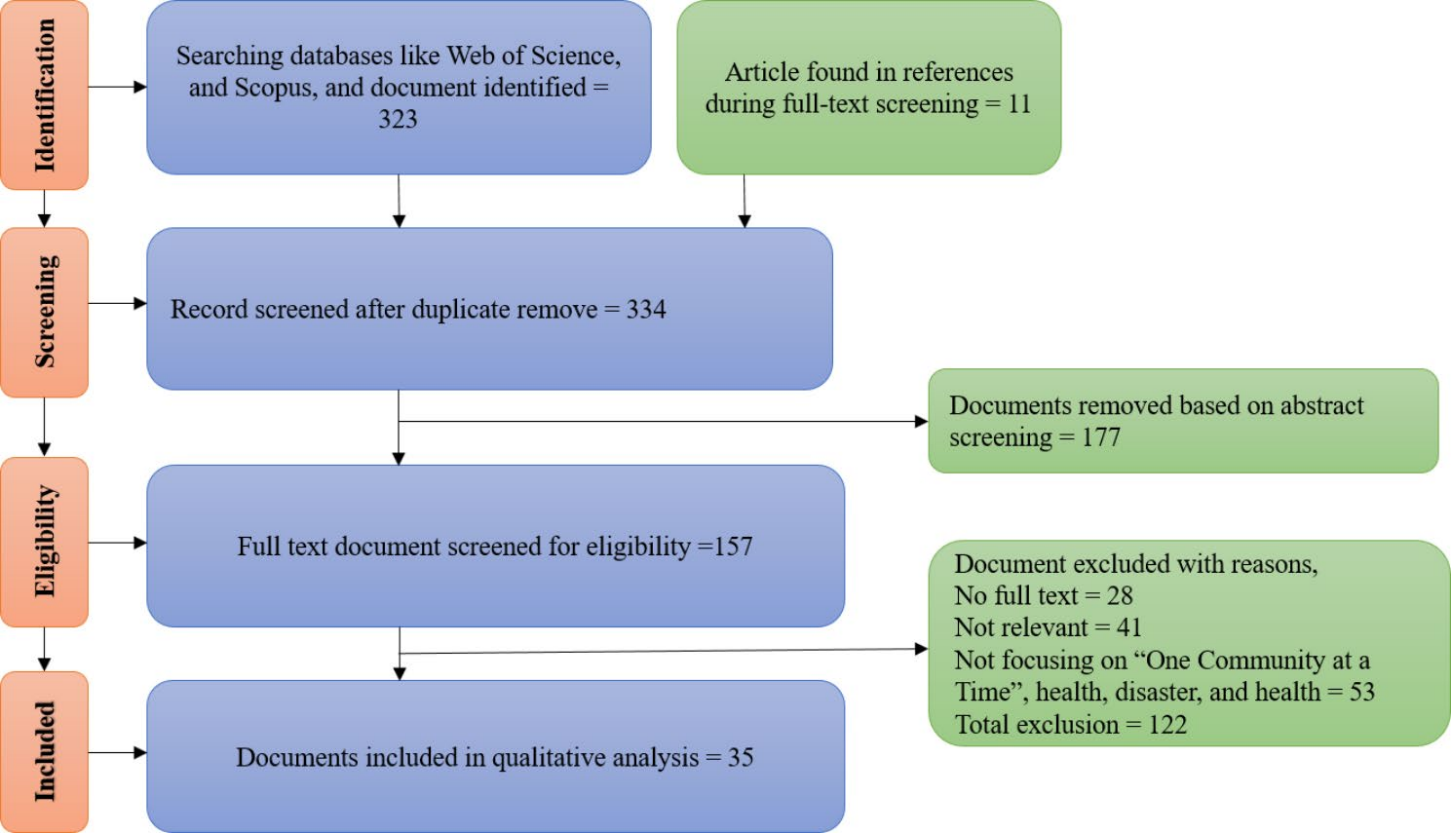
Document selection process by PRIAMA approach

In the initial stage of Identification, 323 documents were retrieved from the Web of Science and Scopus databases, in addition to 11 documents obtained from reference lists. At the screening stage, 177 documents were eliminated based on their titles and abstracts, leaving 334 documents for further examination. In the eligibility stage, 122 documents were eliminated for lack of complete text, lack of relevance to the study, or lack of emphasis on “One Community at a Time”, health, disaster, hazards, and resilience. In the included stage, 35 documents (Supplementary S1) were chosen for qualitative analysis based on their relevance, quality, and degree of relevance to the research question (Fig. 1). Then, these documents were subjected to a comprehensive and systematic analysis, which included classifying, categorizing, and synthesizing the data to identify key themes and patterns.

### Characteristics of included studies

#### Distribution by location

A total of 35 publications were included in the systematic review pertinent to investigating resilience in the face of environmental risks and public health issues. These investigations were geographically dispersed as follows: 12 studies were carried out in North America, 10 in Europe, 6 in Asia, 4 in Africa, and 3 in Oceania. The research represents a wide variety of nations, including both high- and low-income countries.

### Risks and issues facing public health

Numerous risks and issues with public health were covered in the research. Fourteen research specifically addressed floods, eight examined earthquakes, five examined hurricanes or typhoons, and three examined wildfires. Fifteen research examined public health concerns, including infectious illnesses (such COVID-19, Ebola, and influenza) and non-communicable diseases (including chronic ailments and mental health problems). A few research (five) addressed the connection between environmental risks and problems with public health, highlighting the interdependence of these problems.

### Components of “One community at a time”

Five components of “One Community at a Time” framework have been identified in this study that can address the challenges related to disaster-induced public health challenges, and enhance community resilience. The components are social capital and networks, local knowledge and learning, effective governance and leadership, preparedness and response capacity, and adaptive infrastructure and resources. To establish resilience in communities, it is important to understand the function of social capital and networks, which serve as a critical foundation. The local knowledge and learning can help understanding of context and culture contributes to developing resilience measures appropriate for the region. The third component (effective governance and leadership) sheds light on strong institutional structures and capable leadership’s role in fostering resilience. The fourth component (preparedness and response capacity) highlights the value of proactive and adaptable actions in overcoming challenges. The fifth component (adaptive infrastructure and resources) highlights the significance of adaptable physical and economic systems for overcoming obstacles and recovering. Our suggested paradigm for enhancing resilience is built on these five elements (Table 2).

**Table 2:** Components of “One community at a time”

Components	Elements	Relationship with “One Community at a Time”	Sources
Social capital and networks	• Trust and reciprocity • Social cohesion • Bridging and linking networks • Collective action • Informal support networks • Community cohesion • Inter-organizational networks • Online social networks • Civic participation • Community leadership	• Fosters cooperation and mutual support during crises • Strengthens community bonds and resilience • Enhances resource access and knowledge sharing • Empowers communities to address challenges together • Provides emotional, informational, and practical assistance during crises • Enhances community resilience through collective action • Facilitates resource sharing and joint problem-solving • Enhances communication and information sharing • Strengthens community engagement and ownership • Facilitates local decision-making and mobilization	[4, 39] [22, 40] [23, 41] [36, 42] [43, 44] [23, 45] [4, 10] [46] [43, 47] [34, 48]
Local knowledge and learning	• Integration of traditional knowledge • Participatory action research • Capacity building and skill development • Cross-sectoral knowledge exchange • Continuous learning and innovation • Indigenous knowledge integration • Participatory research and planning • Continuous learning and feedback loops	• Enhances community-based adaptation and resilience strategies • Ensures research is grounded in a local context and addresses community needs • Builds local capacity to respond to and manage hazards and challenges • Facilitates knowledge sharing and holistic understanding of hazards and challenges • Encourages ongoing improvement of resilience-building efforts • Enhances local relevance and effectiveness of resilience strategies • Ensures locally tailored solutions and ownership • Supports adaptive management and improvement	[4, 39] [49, 50] [12, 51] [4, 39] [5, 24] [12, 52] [39, 50] [39, 53]
Effective governance and leadership	• Transparent decision-making • Inclusive processes • Adaptive capacity • Collaborative approaches • Long-term perspective • Transparency and accountability • Inclusiveness and equity • Long-term vision and planning • Empowerment and capacity building • Cross-sectoral collaboration • Conflict resolution and consensus building	• Fosters trust and engagement within local communities • Ensures voices of all community members are heard and respected • Facilitates efficient and effective recovery for communities • Supports coordinated efforts at the community level • Encourages future-oriented community development • Builds trust and ensures fairness in governance • Ensures diverse perspectives are considered in decision-making • Supports sustainable resilience-building efforts • Enhances local capacity for resilience-building • Fosters holistic and integrated approaches to resilience • Promotes harmony and cooperation in community efforts	[54, 55] [15, 22] [24, 41, 51] [39, 47, 56] [39, 57] [39, 58] [59, 60] [41, 47] [4, 9] [58] [39, 51]
Preparedness and response capacity	• Early warning systems • Emergency plans and protocols • Resources and infrastructure • Community-level training and drills • Evaluation and learning • Community-based disaster management • Risk communication • Recovery and reconstruction capacity	• Allows for faster community response to hazards • Ensures coordinated and effective community response • Facilitates rapid and effective response during crises • Enhances community preparedness and response skills • Supports continuous improvement of preparedness and response • Encourages local ownership and context-specific approaches • Supports informed decision-making and preparedness • Ensures rapid restoration of community functions	[12, 61] [28, 62] [4, 45] [28, 63] [12, 39] [29, 57] [22, 55] [20, 62]
Adaptive infrastructure and resources	• Climate-resilient infrastructure • Resource allocation and distribution • Green infrastructure and ecosystem services • Technological innovation and adaptation • Financial resources and mechanisms • Decentralized and distributed systems • Adaptive policies and regulations • Knowledge sharing and collaboration	• Minimizes community disruption during natural hazards • Ensures all community members have access to necessary resources • Supports natural systems in providing essential services • Enhances community resilience by leveraging technology • Enables communities to invest in adaptive infrastructure and resources • Enhances community resilience by reducing dependencies • Encourages proactive approaches to resilience-building • Supports learning and dissemination of best practices	[32, 64] [65] [64, 66] [28] [4, 20] [40, 51] [4, 67] [39, 53]

### Framework for building and sustaining community resilience

Initiating the proposed framework for fostering community resilience requires ten key steps, such as assess community vulnerability and risks, engage and mobilize community members, develop a community resilience plan, implement resilience-building initiatives, monitor, evaluate, and revise plans, build partnerships and networks, strengthen local governance and leadership, foster local knowledge and learning, enhance preparedness and response capacity, and develop adaptive infrastructure and resources (Table 3). Several studies have reported that the execution of these steps resulted in a substantial increase in community resilience, and these steps were consistently linked to effective community resilience-building initiatives across various research [23, 29, 47, 56].

**Table 3:** Components of building and sustaining community resilience

Steps for implementation	Possible indicators	Implementation process	Sources
Assess community vulnerability and risks.	• Risk and vulnerability assessments • Hazard maps	Identify hazards and prioritize community needs	[2, 50]
Engage and mobilize community members.	• Number of community meetings • Community member involvement	Build trust and foster participation	[4, 24, 29]
Develop a community resilience plan.	• Resilience plan and goals • Key performance indicators	Create a roadmap for resilience-building	[4, 23, 41]
Implement resilience-building initiatives.	• Number of initiatives • Progress towards goals	Address prioritized needs and vulnerabilities	[8, 47, 56]
Monitor, evaluate, and revise plans.	• Monitoring and evaluation reports • Integration of lessons learned	Continuously improve and adapt strategies	[39, 48]
Build partnerships and networks.	• Number of partnerships • Diversity of partner organizations	Enhance collaboration and resource sharing	[24, 48, 53]
Strengthen local governance and leadership.	• Capacity-building programs • Community leader effectiveness	Enhance community-led decision-making	[5, 58]
Foster local knowledge and learning.	• Training and education programs • Knowledge exchange platforms	Integrate local knowledge and expertise	[12, 52]
Enhance preparedness and response capacity.	• Emergency drills and simulations • Allocation of resources for preparedness and response	Build community ability to respond effectively	[15, 28]
Develop adaptive infrastructure and resources.	• Climate-resilient infrastructure • Investment in sustainable resources	Ensure community infrastructure is adaptive and sustainable	[15, 57, 68]

### Stakeholder roles and responsibilities

The significance of including various stakeholders in fostering and maintaining community resilience was highlighted. The coordination of efforts and resource allocation were shown to be crucially dependent on local government officials, with several studies reporting effective resilience-building projects spearheaded by local governments. Several studies demonstrated how well non-governmental organizations collaborated with local stakeholders, making them important partners in providing technical know-how and assistance [22, 58, 62]. To ensure that the suggested resilience solutions were both culturally acceptable and sensitive to community needs, community members were crucial in guiding the process and providing their local expertise. The findings support the formation and use of a comprehensive framework for fostering community resilience in the face of public health issues and natural hazards. By adopting the specified stages for implementation and including a diverse array of stakeholders with clearly defined roles and responsibilities, communities can successfully improve their ability to respond to and recover from various threats and challenges (Table 4).

**Table 4:** Stakeholder’s roles and responsibilities

Stakeholder’s name	Roles and responsibilities	Relationship with “One Community at a Time”	Sources
Local government	• Develop and implement policies and regulations • Coordinate with other stakeholders	Provide enabling environment and resources.	[28, 47, 69]
Community members	• Participate in planning and decision-making • Implement resilience-building initiatives	Represent local needs and perspectives	[40] [22, 36]
Private sector	• Provide resources and technical expertise • Collaborate on infrastructure projects	Support community initiatives	[47, 53] [56]
Non-governmental organizations (NGOs)	• Support community capacity building • Advocate for community needs and rights	Offer technical assistance and resources	[58, 62] [22]
Media and communication outlets	• Disseminate information and raise awareness. • Facilitate public dialogue and engagement.	Enhance risk communication and knowledge sharing	[46, 55] [56]
International organizations	• Provide funding, technical support, and capacity building • Foster partnerships and cross-border collaboration	Assist in coordinating and scaling up efforts	[50, 53] [39]
Health sector	• Develop public health preparedness plans • Provide emergency health services and resources	Strengthen community health resilience	[70] [63]
Emergency management agencies	• Develop and implement emergency response plans • Coordinate disaster response and recovery efforts	Enhance community preparedness and response capacity	[56] [23]
Faith-based organizations	• Provide emotional and spiritual support • Mobilize resources and volunteers	Address psychosocial aspects of resilience	[15] [48, 52]
Environmental organizations	• Advocate for sustainable practices • Conduct environmental assessments and monitoring	Preserve ecosystems and natural resources	[10, 24] [53]
Educational institutions	• Provide education and training programs • Promote community engagement and participation	Build capacity and enhance community knowledge	[48, 71] [34, 61]
Cultural organizations	• Preserve and promote local cultural heritage • Facilitate intercultural dialogue and understanding	Foster community identity and cohesion	[72, 73] [24]
Youth and community groups	• Engage and empower youth and marginalized communities • Mobilize volunteers and resources	Promote inclusiveness and social equity	[15, 74] [34]

## Discussion

### “One community at a time” paradigm for community resilience

**Fig. 2:**
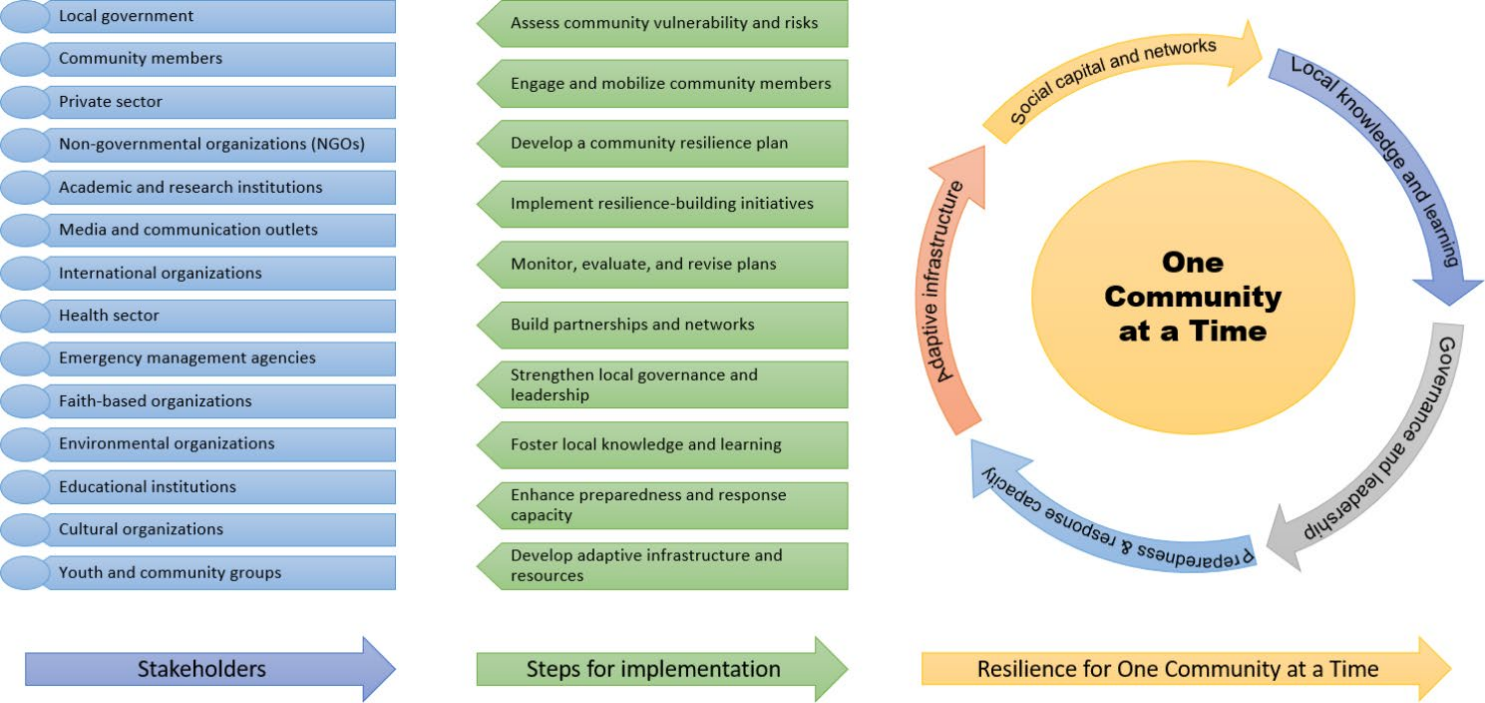
“One community at a time” for community resilience framework

The “One Community at a Time” paradigm provides a comprehensive and integrated framework for enhancing community resilience against public health challenges and natural hazards (Fig. 2). This approach offers a robust strategy for building resilience by encompassing key elements such as social capital and networks, local knowledge and learning, effective governance and leadership, preparedness and response capability, and adaptive infrastructure and resources. A notable strength of this paradigm is its emphasis on participation and community involvement. The integration of social capital and networks within the paradigm facilitates the cultivation of collaboration and cooperation among diverse stakeholders, hence augmenting the community’s ability to effectively and efficiently address and mitigate the impacts of disasters. It recognizes the invaluable insights, experiences, and resources that communities bring, ensuring active community member involvement in the resilience-building process [75, 76]. This approach not only empowers communities to take charge of their resilience but also actively shapes their future through empowerment and mobilization [48].

By incorporating social capital and networks, the paradigm promotes collaboration and cooperation among various stakeholders. Communities can tap into a diverse array of resources, knowledge, and support systems by forging strong connections and networks. Effective coordination and information sharing within these networks enhance the community’s capacity to respond swiftly and adeptly to disasters [77]. Furthermore, networks and social capital cultivate a sense of civic duty and unity, bolstering social cohesion (Fig. 2).

The “One Community at a Time” paradigm acknowledges the unique characteristics and challenges of each community, emphasizing the importance of regional expertise, indigenous traditions, and knowledge gained from past experiences [34]. By valuing and utilizing community-specific knowledge, the paradigm equips communities to develop context-specific strategies and solutions that meet their needs and circumstances. This approach enhances the effectiveness and feasibility of initiatives aimed at fostering resilience. Effective governance and leadership are essential for implementing this idea. The paradigm places a strong emphasis on inclusive decision-making processes that take into account the diverse needs and opinions of community members. Effective governance and leadership enhance the community’s trust and credibility by promoting transparency, accountability, and responsiveness. This, in turn, encourages actions to build resilience and ensures the long-term involvement and commitment of all stakeholders [13].

The paradigm’s focus on preparedness and response capacity addresses the critical need to enhance a community’s ability to withstand and recover from disasters. Investments in early warning systems, disaster preparedness plans, and capacity-building efforts can help communities improve their preparedness and response capabilities. By taking proactive measures, communities can effectively manage disaster risks and mitigate their impacts. Furthermore, the paradigm emphasizes the importance of prioritizing the most vulnerable groups to ensure that resilience-building activities are inclusive and equitable [7].

### Components of “One community at a time”

#### Social capital and networks

A key component of social capital is social cohesiveness, which is the degree to which a community’s members share common values and objectives and have strong interpersonal ties [3]. A cohesive society is more likely to survive the strains of natural disasters and public health issues because it promotes collaboration, facilitates information exchange, and fosters a feeling of belonging and shared responsibility. For communities to prepare for and recover from calamities, social cohesiveness must be encouraged. While connecting networks create connections between community members and outside organizations like governmental bodies, non-governmental organizations, or businesses, bridging networks link various social groupings inside a community [50]. The capacity of community members to cooperate to solve issues and accomplish shared objectives is called collective action, a crucial component of social capital. In the context of resilience-building, collective action can enhance communities’ capacity to prevent, mitigate, and recover from the impacts of natural hazards and public health challenges. By mobilizing resources, coordinating efforts, and sharing knowledge, communities can develop and implement more effective strategies to address emergencies and promote long-term resilience [78].

To create and maintain resilience, community cohesiveness is essential. It entails the efficient operation of social groupings based on interpersonal connections, a sense of shared identity, comprehension, norms, values, trust, cooperation, and reciprocity. As it develops a feeling of collective responsibility and mutual support among community members, stronger community cohesiveness may result in better preparation, response, and recovery in the face of natural disasters and public health concerns. Inter-organizational networks are crucial for fostering community resilience because they enable cooperation and resource sharing across several groups, such as governmental bodies, non-governmental organizations, and businesses [22]. Civic engagement is an essential component of social capital that may support community resilience [3]. It alludes to community people actively participating in decision-making procedures, creating public policy, and undertaking community development projects. High levels of civic engagement may encourage a feeling of ownership and group responsibility among community members, eventually resulting in improved preparation, response, and recovery in the face of natural disasters and public health issues. The building blocks of social capital and resilience are reciprocity and trust. Reciprocity ensures neighbors are motivated to help one another, while trust allows people to depend on others in need. Resilience must be developed and maintained via effective community leadership [22]. Strong leaders may aid during crises by mobilizing resources, coordinating activities, and facilitating communication among community members and external stakeholders.

#### Local knowledge and learning

Traditional knowledge may be used in resilience-building methods to improve community responsiveness to public health issues and natural disasters. Traditional knowledge frequently offers helpful insights into risk management and adaptation techniques since it is founded on local culture and history [74]. By utilizing this information, communities may create context-specific and culturally suitable solutions to their problems. Participatory action research (PAR) is a cooperative strategy that includes people of the community in the research process to pinpoint and resolve regional problems [61]. PAR supports community empowerment by increasing communities’ ability to operate as a unit and choosing ways for resilience-building with knowledge. This strategy encourages community ownership and engagement, eventually leading to more efficient and long-lasting solutions [47]. Collaboration across different sectors, such as the public and corporate sectors, academia, and civil society, may encourage the exchange of knowledge, best practices, and creative solutions, eventually resulting in more efficient and comprehensive approaches to resilience-building [4]. Continuous learning and innovation are essential for adjusting to the constantly evolving nature of environmental hazards and public health concerns. Indigenous cultures often have specialized knowledge of their immediate surroundings and have established tried-and-true methods for overcoming environmental risks and public health issues. By recognizing and using this understanding, resilience-building projects may become more inclusive, context-specific, and successful.

Participatory research and planning guarantee that local knowledge and opinions are considered by including community people in decision-making, encouraging more situation-specific and culturally suitable solutions. The sustained efficacy of resilience-building techniques depends on continuous learning and feedback loops. Communities may pinpoint areas for improvement, alter their strategies, and learn from triumphs and failures by routinely monitoring and analyzing programs. Building resilience in the face of changing environmental dangers and public health issues depends on this process of reflection and adaptation. Communities may create more resilient and adaptable methods that can be improved over time to fit the changing requirements and circumstances of the community by adding continuous learning and feedback loops into the framework [14].

#### Effective governance and leadership

Effective governance and leadership depend on transparent decision-making to develop and maintain community resilience [58]. A trust may be developed between community members and their leaders by ensuring that choices are made honestly and with clear disclosure of the reasoning and supporting data. Through collaborative initiatives, collaboration is promoted amongst many stakeholders, such as governmental bodies, neighborhood associations, and citizens [56]. In addition to fostering a feeling of civic ownership and responsibility for tackling natural disasters and public health issues, these partnerships may result in more thorough and coordinated plans for strengthening resilience. By putting collaboration first, leaders may use their communities’ combined knowledge, abilities, and resources to create more efficient and long-lasting solutions [79]. A long-term viewpoint is essential for resilience-building programs to have a long-lasting effect on communities. To foster and maintain community resilience, transparency and accountability are crucial components of good governance and leadership. Building stakeholder confidence and encouraging shared responsibility are benefits of open communication channels and transparent decision-making procedures.

Inclusivity and fairness are fundamental values in the governance and leadership of community resilience efforts [56]. To guarantee that their opinions are heard, and their needs are met entails actively including various stakeholders in the decision-making process, especially disadvantaged and vulnerable groups. Leaders may guarantee that resilience methods are fairer and more successful, enhancing the community by building an inclusive and just environment. A long-term vision and preparation are necessary for effective governance and leadership in fostering community resilience. This entails proactively spotting new threats and weaknesses and considering any possible long-term effects of current actions. A long-term viewpoint enables leaders to create plans that boost community resilience in the face of upcoming natural disasters and public health issues. To promote community resilience, empowerment and capacity development are essential elements of good governance and leadership. Leaders may empower community individuals to actively engage in projects to create resilience by offering opportunities for skill development, training, and education. This encourages unity and common purpose, allowing communities to collaborate to confront natural disasters and public health issues better [24].

#### Preparedness and response capacity

Early warning systems are essential for community resilience-building and maintenance against natural disasters and public health issues. These systems may assist in identifying new hazards and giving communities timely information so they can take the necessary preventative and mitigation measures [12]. Effective early warning systems must be implemented to reduce the potential effects of risks on public health and community well-being [16]. Effective preparation and response capabilities in the face of environmental threats and public health issues depend on adequate infrastructure and resources. This includes spending on healthcare facilities, communication networks, and other essential infrastructure to assist emergency response activities. The availability of relevant tools, materials, and employees is also essential for prompt and efficient emergency response. Communities may improve their capacity to deal with and recover from public health issues and natural disasters by guaranteeing enough infrastructure and resources. Community-level training and exercises are essential for boosting preparation and response capacity as they improve community members’ knowledge and abilities to respond successfully to natural disasters and public health concerns [62].

A crucial component of preparation and response capabilities is community-based disaster preparedness (CBDP), which gives local people the authority to take control of and participate in disaster risk reduction and management programs [29]. To promote resilience against natural disasters and public health issues, CBDP stresses the value of local expertise, resources, and capabilities. CBDP promotes a feeling of responsibility and guarantees that interventions are customized to each community’s particular needs and conditions by actively including community members in decision-making processes and response activities [80]. Similarly, effective risk communication is essential for preparation and response capability because it aids in informing, educating, and guiding community members during public health crises and natural disasters. Risks should be communicated openly and promptly to build trust, encourage adherence to public health recommendations, and empower communities to decide what is best for their safety and wellbeing. To succeed in various communities, risk communication tactics should also be accessible, sensitive to cultural differences, and flexible to varied audiences. For communities to recover from the effects of natural disasters and public health issues, recovery and rebuilding capability is a critical component of preparation and response.

#### Adaptive infrastructure and resources

Communities may lessen the negative effects of natural disasters and improve their overall resilience by prioritizing the construction and upkeep of climate-resilient infrastructure. Effective resource distribution and allocation are crucial elements of adaptable infrastructure because they guarantee that communities have access to the resources they need to prepare for, address, and recover from natural disasters and public health issues [65]. Building and maintaining resilience in natural disasters and public health issues requires green infrastructure and ecosystem services. Managing stormwater, purifying the air, and regulating temperature is just a few ecosystem services provided by a network of natural and semi-natural regions, or green infrastructure [66]. Communities may use the many advantages of ecosystem services by including green infrastructure in urban design and development. This will increase their capacity for adaptation and lessen their sensitivity to environmental risks and public health issues. Technological innovation and adaptation are also essential for improving adaptable infrastructure and resources [81]. Communities may create creative solutions to handle new risks and problems, enhance their capacity for preparation and response, and increase their overall resilience by using new technology and modifying current ones [5]. As a result, decision-making processes may become more efficient and effective when dealing with natural disasters and problems with public health. Technological improvements can also help stakeholders communicate, share data, and work together.

Adaptive infrastructure and resources must include financial mechanisms and resources because they enable local governments to engage in projects that increase community resilience in the face of public health crises and natural disasters [15]. Communities can obtain sufficient funding to support the creation and upkeep of adaptable infrastructure, resource allocation, and capacity-building efforts by establishing and utilizing a variety of financial mechanisms, such as grants, loans, public-private partnerships, and innovative financing models [47]. Decentralized and distributed systems may dramatically improve community resilience by ensuring that vital resources and infrastructure are dispersed over many sites and minimizing the vulnerability of any single point of failure. Building and maintaining resilience in natural disasters and public health issues depends heavily on adaptive policies and regulations. To help communities successfully adjust to new dangers and difficulties, these rules and laws should be adaptable and sensitive to changing conditions. Communities may make their policies and regulations relevant and efficient in addressing the dynamic nature of environmental hazards and public health issues by integrating adaptive policymaking into their governance frameworks, thereby boosting overall resilience [5].

### Steps for implementation of “One community at a Time”

The recommended processes for executing the “One Community at a Time” framework are developed from the evidence collected throughout the systematic review. The processes include engaging and mobilizing community members in resilience-building endeavors, formulating a comprehensive resilience strategy, establishing collaborative partnerships and networks, and enhancing preparedness and response capabilities. Every individual step plays a critical role in guaranteeing the successful implementation of the framework and enabling communities to develop and sustain resilience when confronted with natural catastrophes and public health crises.

Finding out how vulnerable and risky the community is the first step in putting a strategy for developing and maintaining resilience into action. Identifying possible natural hazards, issues affecting public health, and related dangers may be done by completing thorough hazard and vulnerability assessments [82]. To ensure that the vulnerabilities and hazards identified are relevant and tailored to the community’s requirements, these assessments should consider the community’s particular geographic, socioeconomic, and environmental circumstances. Engaging and organizing locals in resilience-building initiatives is the next phase. This may be done by encouraging community involvement and cooperation and ensuring that all viewpoints are considered throughout the planning and decision-making. Communities may use their pooled knowledge, expertise, and resources to successfully address identified vulnerabilities and dangers by engaging community members, governmental entities, non-governmental organizations, and other pertinent stakeholders [5]. Communities should create a thorough resilience plan detailing precise goals, objectives, and strategies for fostering and maintaining resilience in natural disasters and public health issues based on vulnerability and risk assessments [48].

Building and maintaining community resilience requires the creation of partnerships and networks. Sharing resources, information, and best practices may be easier via collaborative links between regional authorities, community-based groups, academic institutions, and the commercial sector [53]. These alliances may also improve cooperation and coordination when implementing resilience-building programs, eventually providing more successful and long-lasting results. Local communities must have strong governance and leadership to advance efforts to develop resilience [24]. The different needs and viewpoints of community members should be taken into consideration. Local governments should encourage inclusive and participatory decision-making processes. Local leaders may develop trust and credibility by promoting a culture of openness, responsibility, and responsiveness. These traits are essential for maintaining community support for activities to boost resilience and sustain community involvement [48].

Preparation and response capabilities must be improved to lessen the effects of public health issues and natural disasters [15]. Investments in early warning systems, emergency preparedness plans, and capacity-building programs that improve local institutions’ and communities’ capability to react to catastrophes and crises may help accomplish this [28]. Prioritizing the most vulnerable groups will help ensure that efforts to improve preparation and response capability will provide them with the tools and assistance they need to deal with crises and recover. For communities to be resilient over the long term in the face of natural disasters and public health issues, it is crucial to develop adaptable infrastructure and resources. This might include making investments in infrastructure that is climate resilient, supporting green infrastructure and ecosystem services, and using technology innovation and adaptation to meet changing risks and vulnerabilities. Communities may better protect themselves against catastrophes and public health issues by prioritizing investments in adaptable infrastructure and resources while supporting sustainable development and raising general living standards [68].

### Stakeholder roles and responsibilities

Local governments are essential to create and maintain a resilient community facing public health issues and natural disasters [28]. They are responsible for creating and putting resilient policies, rules, and programs into effect, distributing funds to projects that enhance resilience, and coordinating the activities of diverse stakeholders. Local governments should also ensure that residents have access to timely, reliable information and tools to improve their capacity for preparation and reaction [69]. As they have vital local resources, skills, and expertise, community people are important stakeholders in efforts to create resilience. They are accountable for participating in community planning activities, contributing their experiences and viewpoints, and aiding in the execution of measures to promote resilience. Community members should also actively participate in preparation and response activities, such as training, exercises, and educational programs, to improve their ability to deal with natural disasters and public health issues [36].

Public-private partnerships, which enable the transfer of technology, expertise, and resources amongst diverse stakeholders, may also help the business sector participate to resilience-building initiatives [53]. Non-Governmental Organizations (NGOs) are crucial in aiding community resilience-building initiatives by giving resources, advocating for vulnerable people, and offering technical help [62]. They may link local governments and residents, ensuring that attempts to enhance resilience consider vulnerable populations’ opinions and needs. NGOs may also promote capacity-building activities, encourage stakeholder engagement, and assist with implementing, monitoring, and evaluating programs to increase resilience [22]. Academic and research institutions can support the development of community resilience by producing knowledge based on empirical research, creating novel solutions, and offering technical skills to support resilience-building strategies and initiatives [56].

In addition to creating and executing emergency preparation and response strategies, their duties also include organizing the activities of many stakeholders and offering assistance and resources to disaster-affected areas both during and after catastrophes. Emergency management organizations should work with other stakeholders to improve community resilience, response capability, and readiness [23]. They should also make sure that attempts to create future resilience are informed by lessons gained from previous crises. Faith-based groups serve impacted communities during and after natural disasters and public health crises by offering them spiritual, emotional, and material support. This helps to create community resilience.

## Conclusion

This study attempts to present a thorough framework for improving community resilience, focusing on “One Community at a Time”. The framework focuses on the significance of comprehending community vulnerabilities and hazards, mobilizing community members and creating and putting targeted resilience-building programs into practice. Additionally, it emphasizes the obligations of different parties, such as local governments, community residents, businesses, NGOs, academic and research institutions, media and communication outlets, international organizations, the health sector, emergency management organizations, and faith-based organizations. The suggested framework highlights the need for a multi-sectoral, participative, and collaborative approach to developing resilience, highlighting the need for local knowledge, learning, and adaptation in response to changing environmental hazards and public health concerns. It also highlights the importance of monitoring, assessing, and revising strategies for boosting resilience, supporting local government and leadership, and creating networks and collaborations among many stakeholders. By embracing and putting into practice the suggested framework, communities may create and maintain resilience in the face of natural disasters and public health issues, lessening the effects of catastrophes and safeguarding the wellbeing of their citizens. This framework may also act as a foundation for further research and development in resilience-building and a guide for creating context-specific strategies, policies, and interventions to boost community resilience “one community at a time.

### Limitations

Despite using the PRISMA approach for a systematic literature review, the search strategy may have overlooked pertinent research or information sources due to procedural constraints, linguistic limits, or possible publication bias. The generalizability of the suggested framework may also be limited by the fact that each community has its distinct requirements, traits, and capabilities that may call for further modification and personalization. Additionally, while attempts were made to consider the stakeholders’ views, it’s possible that some of their opinions or contributions were not adequately reflected or captured within the framework, indicating the need for more study to fully comprehend the roles and interactions of the stakeholders. Last but not least, it is important to recognize that the idea of resilience is always changing to fresh perspectives, ideas, and methods that may show up in the future, either challenging or improving the framework. By recognizing these drawbacks, future research may expand on this framework and fill in these gaps, improving its application and efficacy in fostering community resilience in natural disasters and public health concerns.

### Research gap and future research direction

There are huge scopes of future studies in the same area, including the need for a deeper knowledge of context-specific resilience approaches, the impact of cultural and socioeconomic elements on community resilience, the role of new and emerging technologies, and the long-term implications of resilience-building activities.

Future studies might examine context-specific resilience strategies for various hazards, considering distinct populations’ unique requirements and vulnerabilities. Researchers should also examine how social and cultural elements interact to shape community resilience and create culturally-sensitive methodologies that draw on regional expertise and traditions. Furthermore, it is important to investigate how cutting-edge technology, such as big data, the Internet of Things, and artificial intelligence, might improve community resilience. Studies conducted over a longer period are required to evaluate the sustainability and long-term efficacy of activities that boost resilience. These subsequent research paths will help us get a more complete and nuanced knowledge of community resilience, which will eventually drive the development of evidence-based policies and practices that will help communities be stronger in the face of natural disasters and problems with public health.

### Electronic supplementary material

Below is the link to the electronic supplementary material.

Supplementary Material 1: Selected documents for in-depth analysis

## Data Availability

Data are available in Supplementary S1.
