# Supplementary material for: “One community at a time”: promoting community resilience in the face of natural hazards and public health challenges
Source: BMC Public Health. 2023 Dec 14;23:2510. doi: 10.1186/s12889-023-17458-x (PMC10722774; doi:10.1186/s12889-023-17458-x)
Supplement: Supplementary file 1 — Supplementary Material 1: Selected documents for in-depth analysis [file 12889_2023_17458_MOESM1_ESM.docx]

**Supplementary S1.** Selected documents for in-depth analysis

| Sl | Author(s) | Objective | Methodology | Key findings | Implications |
| --- | --- | --- | --- | --- | --- |
| 1 | Klein and Coffey [1] | To review the theoretical foundation for community energy and its role in energy behavior change. | Literature review and survey | Community energy initiatives provide a supplementary framework for the implementation of renewable energy sources. | Subsequent investigations are suggested in the expeditiously expanding domain of community energy. |
| 2 | Woolf et al. [2] | To develop and test a framework tool for assessing resilience in slums | Theoretical analysis | Community assets, capacities, and external resources exhibited an enhancement subsequent to the implementation of the framework tool. | The study provides a novel tool for assessing and improving resilience in slum communities |
| 3 | Sharifi [3] | Review tools for assessing community resilience. | Critical review | Identified gaps and limitations in current tools. | Calls for more comprehensive and integrated tools. |
| 4 | Bischoff et al. [4] | Develop disaster resilience indicators. | Literature review and expert consultation | Proposed a set of indicators for disaster resilience. | Provides a benchmark for assessing resilience maturity. |
| 5 | Van Well et al. [5] | Investigate resilience strategies under different flood risk regimes. | Comparative case studies | Identified different resilience strategies based on flood risk management regimes. | Highlights the need for adaptive strategies based on specific contexts. |
| 6 | Kontokosta and Malik [6] | To develop a unified index for benchmarking neighborhood resilience | Quantitative analysis | The Resilience to Emergencies and Disasters Index (REDI) is a valid measure of community resilience. | The study provides a valuable tool for policymakers and agency leaders in resilience planning and emergency management |
| 7 | Dunning [7] | Explore the relationship between various components of coastal governance. | Theoretical exploration | Redundancy can both enhance and reduce resilience. | Calls for a balance between redundancy and complexity in infrastructure systems. |
| 8 | Hermans et al. [8] | Comprehend how EWS conceptualizes the integration of scientific and local knowledge. | Literature review | An integration spectrum between knowledge domains in EWS was identified. | Emphasizes the importance of understanding power dynamics and context in EWS. |
| 9 | Niazi et al. [9] | Assess the psychological resilience of children in the face of adversity. | Questionnaire survey | Significant variations in the psychological resilience of children were identified. | A framework is presented for the evaluation and enhancement of children's resilience. |
| 10 | Gim and Shin [10] | Explore how vulnerability and resilience impact the health of disaster survivors. | Longitudinal analysis | Identified determinants of community resilience that impact wellness. | Highlights the importance of improving community resilience. |
| 11 | Ressler [11] | Explore the intersectionality of social capital and disasters for individuals with SMI. | Theoretical exploration | Identified barriers for individuals with SMI in accessing resources post-disaster. | Provides recommendations for promoting resilience for individuals with SMI. |
| 12 | Ssennoga et al. [12] | Understand the resilience of persons with disabilities in landslide-prone areas. | Cross-sectional design | People with disabilities in landslide-prone areas have low resilience. | Calls for enhancing the resilience of disable people. |
| 13 | Khan et al. [13] | Develop an inclusive disaster resilience index. | IMF index-making methodology | Developed a novel resilience index capturing multiple factors. | Provides recommendations for improving resilience in various countries. |
| 14 | Gerges F, et al. [14] | Propose a hybrid approach for quantifying resilience. | Theoretical exploration | Proposed a metric combining community and infrastructure capitals. | Offers a new approach for measuring resilience. |
| 15 | Berke et al. [15] | Evaluate the degree of coordination and effectiveness in targeting hazardous areas exhibited by networks of local plans. | Assessment conducted in each city by a group of local evaluators | Differences in stakeholder engagement and outcomes between Nashua and Norfolk. | PIRS serves as a prospective paradigm for communities to transition from autonomous resilience initiatives to coordinated strategizing. |
| 16 | Folke [16] | Understand the dynamics of social-ecological systems from a resilience perspective. | Review and analysis | Social ecological systems are analyzed in terms of their nonlinear dynamics, thresholds, unpredictability, and unexpectedness. | Highlights the importance of social processes, knowledge-system integration, leadership, adaptive capacity, and adaptive governance. |
| 17 | Plummer and Armitage [17] | Using complex adaptive systems thinking, construct an evaluation framework for adaptive co-management. | Theoretical exploration | Environmental conditions, livelihood outcomes, and institutional and process conditions are the focal points of this evaluative framework. | Encourages systematic learning and cross-site comparisons in adaptive co-management. |
| 18 | Cutter et al. [18] | Provide a new framework for understanding and assessing community disaster resilience. | Model development | Introduces the disaster resilience of place (DROP) model. | Locally comparative assessments of calamity resilience are intended to be enhanced. |
| 19 | Norris et al. [19] | Introduce a comprehensive framework for understanding resilience that incorporates resource dynamics, stress, adaptation, and wellness. | Theoretical exploration | Associating adaptive capacities with adaptation subsequent to a disturbance, community resilience is defined. | Values community competence, information and communication, social capital, and economic development as adaptive capacities that are emphasized. |
| 20 | Magis [20] | Propose community resilience as an indicator of social sustainability. | Theoretical exploration | Defines community resilience in terms of thriving in a changing environment. | Introduces the Community Resilience Self Assessment as a tool for measuring resilience. |
| 21 | Sherrieb et al. [21] | Measure adaptive capacities for community resilience using population indicators. | Empirical study | Developed a degree of the capacities of community resilience that correlated favorably with established indices. | Provides a foundation for predicting a community's capacity to recover from the risks of disasters. |
| 22 | Felix et al. [22] | As a strategy for health care reform, advocate for community health development. | Theoretical exploration | Emphasizes the importance of community-level planning and engagement for health and human services. | Calls for a national strategy that incorporates community-based experiences. |
| 23 | Davoudi et al. [23] | Explain the resilience concept in the context of climate change adaptation. | Theoretical exploration | Emphasizes adaptation as an ongoing process revolving around social learning and institutional change. | Highlights the need for value-based decision-making in climate change adaptation. |
| 24 | Gaillard and Mercer [24] | Discuss the challenges in translating knowledge into action for disaster risk reduction. | Theoretical exploration | Emphasizes the need for an integrative process involving various stakeholders for effective disaster risk reduction. | Strategies for bridging the divide between top-down and bottom-up approaches to disaster risk reduction are proposed. |
| 25 | Wachinger et al. [25] | Review risk perception literature in relation to natural hazards. | Literature review | Identifies a risk perception paradox where high risk perception doesn't necessarily lead to preparedness. | Provides insights for risk governance and communication strategies. |
| 26 | Johannessen et al. [26] | Improve resilience of WASH systems to hazards through public-private partnerships. | Theoretical exploration | Highlights the importance of ecosystem health and green urban environments for resilience. | Calls for partnerships that support vulnerable groups and promote sustainable management. |
| 27 | Aldrich and Meyer [27] | Explore the significance of social capital in fostering resilience within communities. | Theoretical exploration | Highlights the significance of networks and social capital in disaster recovery and survival. | Provides policy recommendations for strengthening community resilience through social infrastructure. |
| 28 | Gotham and Powers [28] | Analyze the significance of social capital in the process of recovering from a disaster. | Case studies | Communities that possessed resilient social networks demonstrated enhanced capacity to coordinate their recovery endeavors. | Emphasizes the importance of social capital in disaster management and provides insights for disaster management. |
| 29 | Amirzadeh and Barakpour [29] | Construct approaches to enhance the resilience of communities in the face of gradual dangers. | Literature review and case study | Emphasizes the importance of multi-scale approaches to resilience. | Integrated strategies for climate change adaptation and disaster risk reduction are required. |
| 30 | Bhagavathula et al. [30] | Foster collaboration in city governments through capacity building. | Training and competency evaluation | Demonstrates the effectiveness of competency-based training in fostering collaboration. | Recommends long-term strategies to influence administrative structures. |
| 31 | Pretty [31] | In order to investigate the significance of social capital in collective natural resource management. | Review of community-based groups for resource management | Communities can collaborate for long-term resource management through social bonds and norms. | Critical to sustainability and community-based resource management is the significance of social capital, according to the study. |
| 32 | Ching et al. [32] | In order to investigate the intricacies that Puna, Hawaii residents encounter when consecutive natural disasters strike. | Qualitative interviews and participant observation | Themes such as effective information dissemination, application of local and indigenous knowledge, and significant influence on children recurred frequently. | Insights into the reactions of communities and individuals to natural hazards are provided by the study. |
| 33 | Allen [33] | In an effort to determine the extent to which community-based disaster preparedness (CBDP) initiatives can mitigate vulnerability associated with climate change. | Review of CBDP initiatives in the Philippines | CBDP initiatives can both empower and disempower local communities | CBDP should not be considered a panacea for all disaster management issues, the study cautions. |
| 34 | Ronald et al. [34] | In order to reassess the principles underlying emergency planning and community preparedness. | Literature review | The initial goal of attaining emergency preparedness within the community may be compromised in favor of placing too much emphasis on written plans. | To facilitate community readiness, the study provides guidelines for the emergency planning procedure. |
| 35 | Lebel et al. [35] | Determine how societal governance attributes operate to improve the capacity to manage resilience. | Case studies | Adaptive capacity is enhanced through a relationship among knowledge, action, and social-ecological contexts. | The necessity of considering who determines what should be made resilient to what, for whom, and for what purpose, as well as the significance of governance in managing resilience, is emphasized. |
